# *In vitro* Evaluation of the Marginal Fit and Internal Adaptation of Zirconia and Lithium Disilicate Single Crowns: Micro-CT Comparison Between Different Manufacturing Procedures

**DOI:** 10.2174/1874210601812010160

**Published:** 2018-02-22

**Authors:** Francesco Riccitiello, Massimo Amato, Renato Leone, Gianrico Spagnuolo, Roberto Sorrentino

**Affiliations:** 1 Department of Neurosciences, Reproductive and Odontostomatological Sciences, University “Federico II” of Naples, Naples, Italy; 2 Department of Medicine, Surgery and Dentistry, University of Salerno, Salerno, Italy

**Keywords:** All-ceramic restorations, Crown, Zirconia, Lithium disilicate, Glass ceramics, Marginal gap, Internal adaptation, Resin cement

## Abstract

**Background:**

Prosthetic precision can be affected by several variables, such as restorative materials, manufacturing procedures, framework design, cementation techniques and aging. Marginal adaptation is critical for long-term longevity and clinical success of dental restorations. Marginal misfit may lead to cement exposure to oral fluids, resulting in microleakage and cement dissolution. As a consequence, marginal discrepancies enhance percolation of bacteria, food and oral debris, potentially causing secondary caries, endodontic inflammation and periodontal disease.

**Objective:**

The aim of the present *in vitro* study was to evaluate the marginal and internal adaptation of zirconia and lithium disilicate single crowns, produced with different manufacturing procedures.

**Methods:**

Forty-five intact human maxillary premolars were prepared for single crowns by means of standardized preparations. All-ceramic crowns were fabricated with either CAD-CAM or heat-pressing procedures (CAD-CAM zirconia, CAD-CAM lithium disilicate, heat-pressed lithium disilicate) and cemented onto the teeth with a universal resin cement. Non-destructive micro-CT scanning was used to achieve the marginal and internal gaps in the coronal and sagittal planes; then, precision of fit measurements were calculated in a dedicated software and the results were statistically analyzed.

**Results:**

The heat-pressed lithium disilicate crowns were significantly less accurate at the prosthetic margins (p<0.05) while they performed better at the occlusal surface (*p*<0.05). No significant differences were noticed between CAD-CAM zirconia and lithium disilicate crowns (*p*>0.05); nevertheless CAD-CAM zirconia copings presented the best marginal fit among the experimental groups. As to the thickness of the cement layer, reduced amounts of luting agent were noticed at the finishing line, whereas a thicker layer was reported at the occlusal level.

**Conclusion:**

Within the limitations of the present *in vitro* investigation, the following conclusions can be drawn: the recorded marginal gaps were within the clinical acceptability irrespective of both the restorative material and the manufacturing procedures; the CAD-CAM processing techniques for both zirconia and lithium disilicate produced more consistent marginal gaps than the heat-pressing procedures; the tested universal resin cement can be safely used with both restorative materials.

## 1 INTRODUCTION

In recent decades, the patients’ growing demand for highly natural-appearing restorations has led to the development of new all-ceramic materials with improved mechanical characteristics ensuring suitable longevity and limiting technical drawbacks [1-3], which are now replacing traditional metal-ceramic restorations. Nowadays, 2 kinds of all-ceramic dental materials claim to provide optimal mechanical and esthetic characteristics, promising to be suitable for most restorative situations, namely Lithium Disilicate (LD) glass ceramics and polycrystalline zirconium dioxide ceramics [4-6].

LD is a glass ceramic and can be produced by means of both pressable and Computer Aided Design-Computer Aided Manufacturing (CAD-CAM) processing [4-8]. The latter procedure provides standardized and reproducible results reducing the errors deriving from the operator-sensitive variables in the dental laboratory. LD shows good mechanical properties (flexural strength ~350 MPa), has a very appealing translucency and is more suitable than zirconia-based restorations in esthetic areas [1, 6, 7]. It can be veneered with fluoroapatite-based ceramics or used in a monolithic configuration and was initially proposed for clinical use as single crowns (SCs) and 3-unit fixed dental prostheses (FDPs) in anterior regions [4-8].

Zirconia ceramics is a polymorphic and allotropic polycrystalline material produced with CAD-CAM technology from fully or partially sintered blanks [2-5]. It shows excellent mechanical properties (flexural strength ~900-1200 MPa) and improved natural-looking appearance compared to metal ceramics [2-5]. Zirconia is a highly biocompatible metastable material and can hinder crack propagation inducing a remarkable increase in fracture toughness by means of a well-known mechanism called transformation toughening [2-5, 8-11]. The material is usually layered using dedicated veneering ceramics and its favorable clinical performances were extensively investigated on both SCs and 3 and 4-unit FPDs [2-5, 8-15].

The precision of all-ceramic restorations depends on several factors, such as restorative materials, manufacturing procedures, individual characteristics of the prostheses (*e.g*. span length, framework configuration), cementation techniques, effect of veneering and influence of aging [3, 16].

Marginal adaptation is a paramount factor for long-term longevity and clinical success of dental restorations [17-25]. Contour misfit and irregularities may lead to cement exposure to oral fluids, resulting in marginal microleakage and luting agent dissolution. In such conditions, marginal discrepancies enhance percolation of bacteria, food and oral debris, potentially causing secondary caries, endodontic inflammation and periodontal disease [26-29].

Several studies investigated the maximum clinically acceptable marginal gap width and different values were proposed in the literature according to the type of restoration [16-25, 30-33]. For SCs, both LD and zirconia copings showed clinically acceptable accuracy of fit [16, 17, 19, 34-37]. Specifically, maximum marginal openings ranging between 45-120 µm were reported for slip casted and heat-pressed copings [16, 17, 19, 34, 35] while this values decreased to 40-90 µm for CAD-CAM restorations [16, 17, 36, 37]. Nevertheless, a wide range of marginal opening values was described in the literature due to restoration type and location [33].

To date, as there are no clinically consistent evaluation methods, a clear correlation between the precision of fit and the longevity of restorations has not been demonstrated [18, 23]. As a consequence, several marginal gap measurements were defined vertically and horizontally, as well as over- and under-contoured and seating discrepancies [18, 24, 30, 31]. In spite of this, the absolute marginal opening is to date considered the best parameter to evaluate marginal fit as the error at the margin is usually the largest [31].

The best method to measure marginal gaps remains a controversial topic. Several techniques have been described in the literature (*e.g*. direct exploration by means of mirrors and probes, replica technique, light and scanning electron microscopy, micro-computed tomographic evaluation). The most common procedure is the section of restorations and the measurement of the discrepancies under a light or a Scanning Electron Microscope (SEM) [18, 21, 22, 32, 33]; nonetheless, the micro-computed tomography (micro-CT) allows for a non destructive evaluation of the prostheses and is nowadays considered the most updated investigational approach [38, 39].

Several factors may contribute to gap size and seating of a restoration, such as preparation geometry, margin configuration, surface finishing, manufacturing system, type of cement, cement layer thickness, cementation technique and pressure [18, 40]. Furthermore, as with CAD-CAM systems, the accuracy of fit may be influenced by scanning procedures, software design, milling procedures and shrinkage compensation [22, 38, 41].

Both manual and computerized die spacing allow for cement thickness compensation during manufacturing procedures, in order to improve the fit of the copings [18, 42]. The thickness of the luting agent should be as thin and uniform as possible [18, 43], as its increase could result in reduced fracture strength of all-ceramic restorations [22, 44]. Moreover, excessive cement space was proved to be related to chipping of the veneering ceramics [45]; consequently, most studies suggested die spacing between 30-50 µm [18, 46].

To date, both LD ceramics and polycrystalline zirconia can be cemented with resin luting agents; resin cements provide favorable mechanical characteristics (*i.e*. good compression strength, low solubility, good wear resistance) and good esthetics [46-50].

The aim of the present *in vitro* study was to evaluate the marginal fit and internal adaptation of zirconia and lithium disilicate single crowns, produced with different manufacturing procedures and cemented with a universal resin cement, by means of a non-destructive approach using micro-CT analysis.

The following null hypothesis was tested: there is no difference in accuracy of fit among the restorations produced with different materials and fabrication techniques.

## 2 MATERIALS AND METHODS

Forty-five human intact maxillary premolars extracted for periodontal reasons were selected for the study; teeth with caries and/or previous restorations were excluded. Dental plaque, calculus and periodontal tissues were carefully removed using hand and ultrasonic instruments. The teeth were stored in 0.5% chloramine T solution at 4°C to prevent bacterial growth.

### 2.1 Tooth Preparation and Sampling Procedures

An expert prosthodontist carried out the preparations for single crown restorations using diamond rotary burs with calibrated diameters under constant water cooling. Silicon indexes were obtained from the unprepared teeth and used to check preparation depths [51-53]. The preparations were standardized as follows:

- margin design: 1 mm circumferential rounded chamfer, with rounded cavosurface angles to prevent stress concentrations;
- axial reduction: 1.5 mm;
- occlusal reduction: 1.5-2 mm (anatomically shaped);
- total occlusal convergence angle: 12°.

The cervical margins were placed in enamel and followed the cemento-enamel junction; then they were polished with fine and extrafine diamond burs. The preparations were finally checked by means of a digital caliper with a precision of 0.01 mm.

The prepared teeth were duplicated by means of a polyvinylsiloxane impression material (Elite Double, Zhermack, Badia Polesine, Italy) and master casts were then prepared with type IV extra-hard stone (Fujirock Pastel Yellow, GC, Tokyo, Japan) strictly following the manufacturers’ mixing and pouring recommendations.

The specimens were placed in sealed opaque envelopes and randomly distributed into 3 groups (n=15) as follows, according to the type of crown they would subsequently receive:

- group 1: CAD-CAM zirconia single crowns (Katana Zirconia, Kuraray Noritake, Tokyo, Japan);
- group 2: CAD-CAM LD single crowns (IPS e.max Cad, Ivoclar Vivadent, Schaan, Liechtenstein);
- group 3: heat-pressed LD single crowns (IPS e.max Press, Ivoclar Vivadent).

All the laboratory procedures were performed by the same expert dental technician.

### 2.2 CAD-CAM Manufacturing Procedures (Groups 1 and 2)

The CAD-CAM manufacturing for both zirconia and LD was done with the CARES System (Straumann, Basel, Switzerland). Each master cast was precisely positioned in the holder of the scanner according to the manufacturer’s indications and the scanning output was carefully verified.

The CAD process was differentiated according to the material to be used according to the specifications of the dedicated software (CARES Visual 6.2).

The CAD of the copings started by choosing the most appropriate shape of the entire final crown from an anatomic library. Then, a uniform layer was virtually cut back from the outer surface of the crown, resulting in an improved customized coping design to provide adequate support to the veneering ceramics. The CAD file was sent to a centralized milling center to complete the CAM procedures and the copings were received after 5 working days. A stereomicroscope (OPMI PROergo, Zeiss, Oberkochen, Germany) at 24x magnification was used to verify the correct fit of the copings on the relative master casts. The zirconia copings were sandblasted with 100 µm aluminum dioxide particles (Cobra, Renfert, Hilzingen, Germany) at a pressure of 1.5 bar. Differently, the LD copings were subjected to a crystallization process in a specific dental oven (EP 3000, Ivoclar Vivadent) with a dedicated heat-pressing program with the following parameters:

- starting temperature: 700°C;
- heating temperature: 60°C;
- final temperature: 920°C.

Each coping was kept in the oven at the final temperature for 25 min.

The milling pins were carefully removed with a diamond rotary disc under constant water cooling so as not to heat the ceramics. Finally, the LD copings were sandblasted with 100 µm aluminum dioxide particles (Cobra, Renfert) at a pressure of 1.5 bar, while a liner (GC Initial, IQ-LOZ, GC) was applied onto the zirconia copings in order to enhance the esthetic performance and the bond between them and the veneering ceramics.

### 2.3 Heat-Pressing Manufacturing Procedures (Group 3)

In group 3, the heat-pressed LD single copings were fabricated strictly following the manufacturer’s instructions. Two layers of die spacer (Die Spacer Dentin, Renfert) were applied on each master cast; once they dried, an additional layer of an isolating medium (Isolit, DeguDent, Hanau, Germany) was applied. The abutment of each master cast was dipped in a specific device (Hotty, Renfert) creating a wax (Geo Dip Green, Renfert) coping with uniform thickness, precise and free of tensions. Such copings were anatomically shaped with a modeling wax (Thowax Beige, Yeti Dental, Engen, Germany) in order to properly support the veneering ceramics. A sharp blade was used to remove 1 mm of the modeling wax circumferentially from the cervical margin; then, this area was sealed with a specific cervical closure wax (Inlay Wax Soft Violet, GC) using a stereomicroscope at 24x magnification. The specimens were placed in the center of prefabricated molds and then invested with a rapid medium (Press Vest Speed, Ivoclar Vivadent) according to the manufacturer’s recommendations. The LD pellet (IPS e.max Press MO1 Ingot, Ivoclar Vivadent) was selected and put in a specific dental oven (EP 3000, Ivoclar Vivadent) with a dedicated heat-pressing program with the following parameters:

- starting temperature: 700°C;
- heating temperature: 60°C;
- final temperature: 920°C.

Each coping was kept in the oven at the final temperature for 25 min.

The refractory material was cleaned by sandblasting the specimens with 50 µm glass beads at 4 bar pressure (Rolloblast, Renfert); further polishing was performed reducing the sandblasting pressure at 2 bar using a stereomicroscope at 24x magnification for better control. Then, the surface reactive layer due to heat-pressing was removed by means of an ultrasonic bath with <1% hydrofluoric acid (Invex Liquid, Ivoclar Vivadent). The molding pins were carefully removed with a diamond rotary disc under constant water cooling, in order not to heat the ceramics. Finally, each coping was sandblasted with 100 µm aluminum dioxide particles (Cobra, Renfert) at a pressure of 1.5-2 bar.

### 2.4 Veneering Procedures

All the copings were anatomically veneered with dedicated ceramics using a layering technique; the silicone indexes used to perform calibrated tooth preparations were employed to check ceramic thickness as well, respecting the initial anatomical shape of each specimen. The crowns were veneered and glazed one next to the other, in order to achieve the most similar final shapes.

The firing was done in the above mentioned dental oven strictly following the manufacturer’s instructions. Finally, all the crowns were finished and polished with stones and silicone points (Porcelain Adjustment Kit HP and Porcelain Veneer Kit HP, Shofu, Inc., Kyoto, Japan).

### 2.5 Cementation Procedures

All the experimental crowns were cemented using a dual cure universal resin cement (Panavia V5, Kuraray Noritake), strictly following the cementation procedure suggested by the manufacturer.

As to the zirconia restorations (Group 1), the inner surface of each crown was conditioned by means of the Ceramic Primer Plus for 30 sec; conversely, as to the LD restorations (Groups 2 and 3), the inner surface of each crown was etched with hydrofluoric K-etchant for 5 sec, thoroughly rinsed and dried and conditioned by means of the Ceramic Primer Plus for 30 sec. In all the experimental groups, the teeth surfaces were treated with the Tooth Primer that was applied, left for 20 sec and thoroughly dried with mild air.

Both for zirconia and LD crowns, the automixed cement was dispensed on the inner surface of the restorations and the crowns were carefully seated onto the abutments with finger pressure; cement excesses were carefully removed by means of microbrushes; then, light curing was performed for 3 sec on each surface of the crowns to gelify minor remnants of cement that were carefully removed with a plastic curette using a stereomicroscope at 24x magnification.

**Fig. (1):**
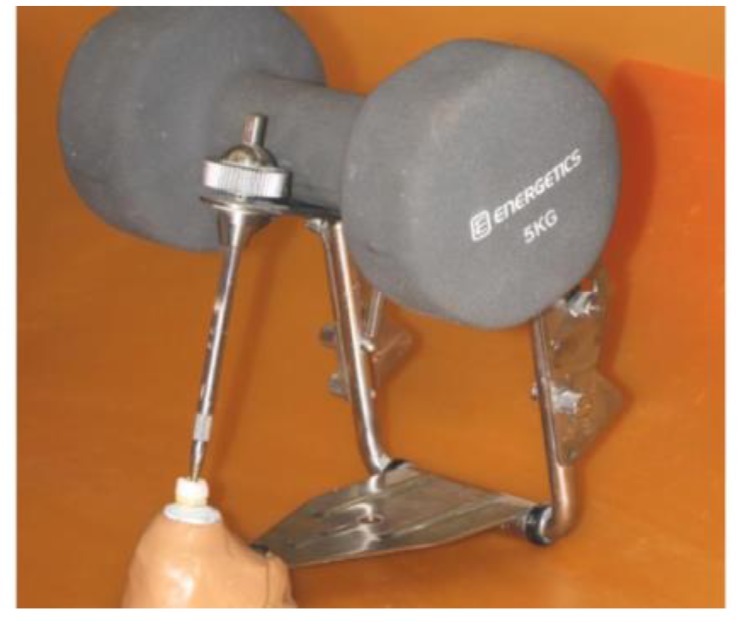
A constant pressure of 5 Kg was applied onto each crown until polymerization of the cement was complete. The restorations were positioned on the plate of an articulator and a 5 Kg weight was kept on the upper arm of the device.

Luting procedures were performed under a constant pressure of 5 Kg until polymerization of the cement was complete (Fig. **1**). The material was left to self-cure for the first 5 min and then additional light-curing polymerization with a glycerin barrier was performed on each crown surface for 40 sec.

All specimens were stored in a laboratory oven at 37°C and 100% relative humidity for 24 hours and then prepared for precision of fit analysis.

### 2.6 Precision of Fit and Micro-Computed Tomography (Micro-CT) Analysis

The median sections in the coronal (mesial-distal direction) and sagittal (buccal-palatal direction) planes of each specimen were identified by means of a micro-CT scanning (Skyscan 1072, Bruker, Kontich, Belgium) and analyzed in a dedicated software (NRecon 1.6.6.0, Bruker) for the evaluation on marginal and internal fit.

**Fig. (2):**
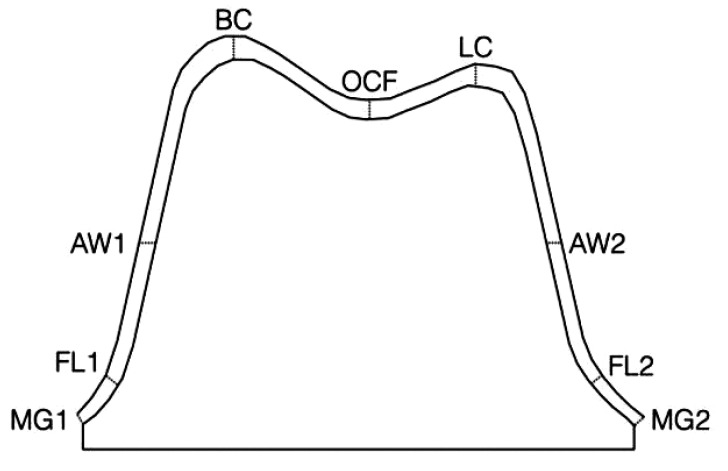
Anatomical distribution of the reference points used for micro-CT measurements.

Eighteen measuring locations (9 per section) were used to evaluate the cement thickness in µm along the entire preparation, in order to assess both the marginal opening and the internal fit of the crowns [22] (Fig. **2**, Table **1**). The measurements were obtained in µm from each surface of the specimens, particularly from the buccal and palatal surfaces in the sagittal section and from the mesial and distal surfaces in the coronal section; the measuring locations were summarized in Table **1**.

Each tooth was scanned using micro CT (SkyScan 1072, Bruker) with the following settings: 10 W, 100 kV, 98 mA, a 1 mm thick aluminum plate, 15x magnification, 4.9 s exposure time and 0.45° rotation step. The acquisition procedures consisted of the creation of several 2D lateral projections of the specimens during a 180° rotation around the vertical axis. The digital data were elaborated using a reconstruction software (NRecon 1.6.6.0, Bruker), which provided new axial cross-sections with a pixel size of 19.1x19.1 µm; the distance between each cross-section was 38.0 µm and the cross sections were collected for each sample. The pixel-micron conversion ratio and the cross-section distance were set in measurement tools included in a dedicated software (Mimics 12.1, Materialize, Leuven, Belgium), thus areas and volumes were calculated. Images were acquired from 972 slices of each tooth. After cone beam reconstructions, the raw data were converted into 16-bit dynamic grayscale picture files with a 2000x2000 pixel bitmap (BMP) format and saved in a specific program (Skyscan Data Viewer 1.4.4, Bruker) to complete the reconstructions. The cementation areas were reconstructed three-dimensionally and a semiautomatic threshold-based segmentation approach was combined with manual editing of slices.

Two calibrated investigators, who had been blinded with regard to the experimental groups, independently measured the precision of fit of the specimens. The measurements were repeated 3 times and the values averaged. Intra-examiner reliability was assessed by the Kappa test (K=0.88).

### 2.7 Statistical Analysis

The data were statistically analyzed using a statistical software (SPSS 16.0, SPSS Inc., Chicago, IL, USA).

Due to the pooled data set of cement thicknesses measured in the 3 groups at different levels, the adaptation values at measuring locations were analyzed by means of One-Way Analysis of Variance (ANOVA) followed by the Tukey's test for post-hoc comparisons as needed, in order to compare cement thicknesses at different levels within the same group and between different groups at the same level.

The absolute marginal opening was calculated as the sum of the measurement locations MG1-2 and FL1-2 while the internal fit was calculated as the sum of the measurement locations AW1-2, BC, OCF and LC.

In all the statistical analyses, the level of significance was set at *p*<0.05.

## 3 RESULTS

Mean values and Standard Deviations (SD) recorded at all measuring locations in each group are reported in Table **2**.

Descriptive statistics of absolute marginal opening and internal fit measurements relative to crown type were shown in Table **3**, along with significant differences according to the One-Way ANOVA and post-hoc test.

When thicknesses of the luting agent were compared at different levels within each group and between groups, the cement layer was thicker at the occlusal wall and thinner at the preparation margins.

As regards crown type, the heat-pressed LD copings were significantly less accurate at the preparation margin (*p*<0.05) while they performed significantly better at the occlusal level (*p*<0.05). Although no statistically significant differences were evident between zirconia and LD (*p*>0.05), CAD-CAM zirconia crowns showed the best marginal adaptation.

## 4 DISCUSSION

On the basis of the results of the present study, the null hypothesis was rejected since the heat-pressed LD crowns were significantly less accurate than CAD-CAM zirconia and LD crowns at the preparation margins.

The long-term success of dental restorations depends on the mechanical and bonding properties of the restorative materials [24]. Consequently, crowns longevity is strongly related to the quality of marginal fit [17-25]. Moreover, poor internal fit of a coping may increase cement thickness negatively influencing the mechanical stability of a prosthesis [24, 44]. To date, marginal openings of no more than 100 µm are considered clinically acceptable [17, 18, 22-24, 30-33, 43].

The marginal gap of zirconia single crowns was reported to range between 36.56 µm and 70.94 µm [37, 54], whereas the precision of fit of lithium disilicate restorations varied between 61.86 µm and 103.75 µm [38, 54]. A recent literature review reported that there is no consensus regarding the precision of fit of different crown systems because of differences in experimental protocols and testing approaches; although the direct view technique was the most common research method, the use of at least 50 measurements per specimen and the combination with micro-CT analysis should carry out more reliable results [35]. Similarly, another systematic review pointed out that the current state of the literature does not allow for a detailed comparison of different restorative systems in terms of marginal fit and the use of micro-CT should be recommended [19].

Although sample sectioning and light or SEM evaluation have been used for years to evaluate the marginal and internal fit of restorations [18, 21, 22, 32, 33], it is worth noticing that those approaches are destructive methods that can be performed on a limited number of tooth slices and sectioning inevitably involves the loss of some information; furthermore, the cutting procedures are time-consuming and preclude further use of the specimens [55].

Recently, the microCT analysis has been proposed for the assessment of marginal leakage and precision of fit of dental restorations. Its main advantage is to provide an uninterrupted visualization of the tooth-restoration interface in a non-destructive way, with further possibility of qualitative and quantitative structure analysis and 3D reconstructions [56, 57]; moreover, the microCT approach was reported to be a reliable and more effective alternative to the traditional sectioning methods [58]. A drawback of the sectioning technique is that analyses.

According to such a scientific background, the micro-CT approach was chosen in the present investigation, although a very few papers are to date available in the literature [19, 35, 38, 39]. This innovative method allowed for the rotation of the samples in the radiological beam to evaluate both qualitatively and quantitatively the entire cementation areas and radiographs were taken at discreet intervals; then, the softwares reduced these radiographs to tomograms which were sliced through the sample on the axis of rotation; consequently, the tomograms could be viewed as 3D blocks of data, allowing for a reliable evaluation of the precision of fit of restorations. The results of the present analysis were in accordance with previous investigations [38, 39]. The marginal gap can be defined as the perpendicular measurement from the internal surface of the margin of the crown to the outermost edge of the finish line of the tooth margin. Similarly, the internal gaps can be defined as the perpendicular distances from the internal surfaces of a coping to the axial and occlusal walls of a preparation [31].

Besides manufacturing procedures and material-related variables, the precision of fit is mainly related to the cement thickness, as during cementation this space will be filled with cement; according to previous investigations, the amount of internal relief and resulting tightness was controlled with the cement space thickness setting of the CAD software; the virtual spacer was set at 30 μm. After the copings were fabricated, the fit of the crowns was carefully evaluated in the dental laboratory by means of a stereomicroscope at 24x magnification and no evident gaps were noticed [3, 16, 18, 22, 38, 40, 41, 54]. The accuracy of a crown is best when the least amount of cement is used at the margins and axial walls [22, 43, 44]. The cement space should be uniform and facilitate seating without compromising retention and resistance of the crown [43, 44].

Several studies were performed to evaluate the cement thickness resulting from different crown/cement configurations [32-36, 38, 40, 43, 44, 46, 48]. The marginal discrepancies varied considerably based on external or internal evaluations [31]. It is worth mentioning that several studies pointed out that the initial results showed lower gaps than those recorded after cutting the samples and observing the internal adaptation [31, 59]. In the present study, the sample size and the number of measurements per crown were selected in accordance with previous investigations [17, 22, 24, 35, 60]. The chamfer preparation was selected as no significant differences were reported in the literature regarding the influence of finish line type on the accuracy of fit assessments [37, 60-62].

As reported in previous investigations, the mean values of the present study demonstrated large SDs, due to the high variation of accuracy within any crown system [17, 63, 64]. Particularly, local adaptation values may be influenced by the asymmetric anatomical shape of the copings as well as by non-uniform distortions during porcelain firing [17, 63, 64].

The results of the present *in vitro* investigation were consistent with those achieved in similar previous studies [17, 18, 20, 22-25]. All the tested crown configurations showed clinically acceptable values of marginal discrepancy. Particularly, the CAD-CAM zirconia crowns showed the best precision of fit at the preparation margins. This was probably due to the fact that dental CAD-CAM systems were developed to process polycrystalline materials and, consequently, the dedicated software produce better blanks [18, 20, 22, 23]. Although they are already clinically acceptable, further developments in dental CAD-CAM technologies would probably improve the performances of such systems in the manufacturing of glass-based materials such as LD-based ceramics, since possible errors may be compensated in different processing steps [23].

Many manufacturers recommend the milling of zirconia in presintered blanks, although sintering shrinkage may negatively influence the precision of fit [1-6, 18, 22, 23]; nonetheless, to date, there is no evidence that milling fully sintered zirconia blanks provides superior marginal fit [3, 23]. Moreover, compensatory software features are nowadays available to avoid such a problem [23]. According to the results of the present study, the CAD virtual spacing proved to be effective in providing adequate space to accommodate the cement.

Regarding the internal fit of the restorations, no differences were observed at axial walls while at occlusal level the heat-pressed LD crowns showed a better adaptation than CAD-CAM processed crowns; moreover, previous studies also suggested that the adaptation of CAD-CAM restorations is less accurate in internal areas [19, 20, 22-24, 37, 54]. The space between the occlusal wall of a preparation and the internal surface of a crown works as a chamber to allow a good marginal adaptation of the crown itself. It could be speculated that the better performance of group 3 crowns at this level was due to the residual thermal stresses deriving from porcelain firing that allowed the heat-pressed LD cores to shrink towards the center of their mass [1-6]; conversely, such a phenomenon was absent in the CAD-CAM manufactured restorations.

The present *in vitro* investigation was a preliminary evaluation of the marginal and internal adaptation of different types of all-ceramic crowns. Experimental studies with thermal cycling and cyclic loading stress protocols would be desirable to confirm the results of the present study.

Furthermore, although the *in vitro* recorded gaps are in accordance with current clinical parameters, *in vivo* adaptation values could be far higher. Consequently, randomized clinical trials evaluating the tested crown-cement configurations would be necessary to substantiate the clinical outcome in the medium-long term.

## CONCLUSION

Within the limitations of the present *in vitro* investigation, the following conclusions can be drawn:

- All the tested crown systems showed clinically acceptable marginal discrepancies;
- Both zirconia and lithium disilicate CAD-CAM crowns showed better marginal adaptation than the heat-pressed lithium disilicate crowns;
- The universal resin cement showed good precision performances irrespective of the restorative material.

## Figures and Tables

**Table 1:** Reference points used for micro-CT measurements.

**Legend Abbreviation**	**Measurement Location**
MG1	Marginal Gap Buccal/Mesial
FL1	Finish Line Buccal/Mesial
AW1	Axial Wall Buccal/Mesial
BC	Buccal Cusp
OCF	Occlusal Central Fossa
LC	Lingual Cusp
AW2	Axial Wall Palatal/Distal
FL2	Finish Line Palatal/Distal
MG2	Marginal Gap Palatal/Distal

**Table 2:** Mean values (±SD) of coping fit (in µm) at the experimental measurement locations.

**Measurement Location**	**CAD-CAM Zirconia**	**CAD-CAM Lithium Disilicate**	**Heat Pressed Lithium Disilicate**
	**Mean Value (±SD)**	**Mean Value (±SD)**	**Mean Value (±SD)**
**MG1**	63 (±32)	65 (±17)	89 (±42)
**FL1**	71 (±16)	69 (±20)	86 (±18)
**AW1**	75 (±21)	71 (±18)	97 (±26)
**BC**	111 (±21)	124 (±32)	99 (±16)
**OCF**	116 (±32)	123 (±25)	91 (±39)
**LC**	125 (±25)	129 (±31)	108 (±32)
**AW2**	76 (±25)	74 (±24)	88 (±14)
**FL2**	79 (±14)	71 (±24)	84 (±26)
**MG2**	69 (±33)	68 (±36)	82 (±22)

**Table 3:** Statistical analyses (One-Way ANOVA and Tukey’s post hoc test for multiple comparisons) of absolute marginal opening and internal fit values among the experimental groups; different letters indicate statistically significant differences (*p*<0.05).

–	**Absolute Marginal Opening**	**Internal Fit**
**Group 1 (CAD-CAM Zirconia)**	65 (±23) A	117 (±44) A
**Group 2 (CAD-CAM Lithium Disilicate**	69 (±41) A	125 (±28) A
**Group 3 (Heat Pressed Lithium Disilicate)**	85 (±26) B	99 (±27) B
